# Vestibular assessment in children aged zero to twelve years: an integrative review

**DOI:** 10.1016/j.bjorl.2022.09.006

**Published:** 2022-10-14

**Authors:** Danielle Samara Bandeira Duarte, Anna Marial de Lira Cabral, Diana Babini Lapa de Albuquerque Britto

**Affiliations:** a Universidade Federal de Pernambuco (UFPE), Recife, PE, Brazil; b Universidade Federal de Pernambuco (UFPE), Curso de Fonoaudiologia, Recife, PE, Brazil; c Universidade Federal de Pernambuco (UFPE), Departamento de Fonoaudiologia, Recife, PE, Brazil

**Keywords:** Vestibular evaluation, Vestibular alterations, Vertigo, Infants, Children

## Abstract

**Objective:**

To describe the main vestibular assessment tests performed in children aged zero to twelve years and the main causes of referral for vestibular assessment.

**Methods:**

The review was guided by the following question: What are the main vestibular assessment tests performed in children aged zero to twelve years and the main causes of referral for vestibular assessment? The PVO strategy was used, being defined as: Population (P) – newborns and children; study Variable (V) – causes of referral for vestibular assessment; study Outcome (O): the main vestibular assessment tests and the main findings. This study was carried out using the main available databases in the months of July, August and September 2021, with no restrictions regarding language and publication date, namely: PubMed, Web of Science, Scientific Electronic Library Online (SciELO), Latin-American and Caribbean Literature in Health Sciences (LILACS), ScienceDirect, Cochrane Library and Embase. The descriptors were obtained from the MeSH database: newborn, infant, child, children, vestibular screening, vestibular infant screening, vestibular newborn screening, test, vestibular function, vestibular function test.

**Results:**

A total of 7,078 studies were identified. After reading the titles and abstracts, 107 of them were selected, with 101 remaining after the exclusion of duplicates. After the full-text reading, 31 articles were included. It was observed that the most frequently used tests were: rotational tests, caloric stimulation and cervical vestibular evoked myogenic potential and the main causes of referral for vestibular evaluation were hearing loss and vestibular screening.

**Conclusion:**

The main tests for vestibular screening and/or assessment of children aged zero to twelve years are the rotary chair testing, caloric stimulation and cervical-vestibular evoked myogenic potential. Consequently, performing these procedures is extremely important, since the presence of vestibular dysfunction is quite common in the studied population.

## Introduction

For a good body balance, the joint action of the visual, locomotor and vestibular system activities is crucial, as well as the integration of these systems’ activity with the central nervous system (CNS).^1^ When conflicting information occurs in one or more of the aforementioned systems, dizziness and body instability are the symptoms that usually appear in the affected subjects.^2^

A child with vestibular dysfunction can also show alterations in communication skills, changes in cognitive impairment, psychological disorders such as social isolation, as well as poor school performance. Moreover, most of the time, children do not know how to report or describe the body changes caused by such dysfunctions.^3^, ^4^

Vestibular screening is the most adequate way to identify early changes related to balance in newborns (NB). As children grow, the neurotological assessment becomes more complete and can be performed with more complex and age-appropriate tests.^2^, ^5^

Since the 1980s, there has gradually been a growing awareness of possible vertigo syndromes in the pediatric population and, as a result, the need to increasingly understand about vestibular function in this age group has emerged.^6^

The area of ​​vestibular assessment in children has become popular in audiology and otorhinolaryngology clinics in recent years. If there is a slightest suspicion of vestibular involvement, due to any suspicious symptomatology, the child should be referred for appropriate neurotological evaluation, in an attempt to establish a correct diagnosis and then initiate a more appropriate treatment, if necessary.^7^, ^8^

Similarly to the early recognition of hearing impairment, the early identification of vestibular disorders has been developed in the pediatric population, since the earlier the identification, the earlier intervention strategies can be implemented.^7^ It is important to note that a good choice of vestibular tests is necessary, as most tests are based on visual motor skills that are only fully developed by 14 to 18 years of age.^6^, ^9^

Researchers, audiologists and physicians have increasingly contributed with valuable information both about vestibular disorders in the pediatric population and in relation to vestibular assessment techniques that are useful for children.

The present integrative review aimed to describe the main vestibular assessment tests performed in children aged zero to twelve years and the main causes of referral for vestibular assessment.

## Methods

This study is an integrative review, which was guided by the following question: What are the main vestibular assessment tests performed in children aged zero to twelve years and the main causes of referral for vestibular evaluation? The study was carried out in July, August and September 2021, through a search in the main available databases, namely: PubMed, Web of Science, Scientific Electronic Library Online (SciELO), Latin-American and Caribbean Literature in Health Sciences (LILACS), ScienceDirect, Cochrane Library and Embase.

There were no language and publication date restrictions. To obtain a larger number of articles, a search key associated with the Boolean operators AND and OR was used, namely: (newborn OR infant OR child OR children) AND (vestibular screening OR vestibular infant screening OR vestibular newborn screening OR test, vestibular function OR vestibular function test). All descriptors used in the search keys were obtained from the Medical Subject Headings (MeSH) database.

### Search strategy

The PVO strategy was used, being defined as follows: Population (P) ‒ newborns and children; study Variable (V) – causes of referral for vestibular evaluation; study Outcome (O): the main vestibular assessment tests and the main findings.^10^

### Selection criteria

Two independent reviewers initially selected the articles by reading the title and abstract and, finally, by reading the full-text article, according to the pre-established inclusion and exclusion criteria. Discrepancies regarding study selection and data extraction were discussed between the reviewers at the end of each step, aiming to reach a consensus, and in the absence of agreement, a third evaluator was consulted.

The studies were included without restrictions regarding the design type, as follows: (1) Description of human patients, either newborns and/or children, aged between zero and twelve years, submitted to any type of vestibular evaluation and/or or self-perception questionnaire related to vestibular function. The exclusion criteria adopted for the review were: (1) Literature review; (2) Book chapters; (3) Studies involving children with vestibular disorders of neurological origin; (4) Course Completion Works, dissertations and theses; (5) Animal studies.

### Data analysis

The reviewers independently extracted data from the selected articles in digital format, namely: article title, authors' names, year of publication, country, type of study, study objective, sample size, age range of the studied group, performed exams, vestibular alterations, complaints for referral, main conclusions provided by the studies and level of evidence. Aiming to synthesize the information from the articles, the data extracted from the studies were descriptively recorded into a previously prepared table, which facilitated the identification and reformulation of thematic categorizations.

The studies were also classified based on the new evidence pyramid, where studies are classified into five levels: level one (systematic reviews/meta-analysis), level two (randomized clinical trials), level three (non-randomized clinical trials), level four (observational studies) and level five (case studies/reports).^11^

## Results

A total of 7,078 studies were identified in the initial search, of which 107 were selected after reading the titles and abstracts. Of the 107 studies selected by reading the titles and abstracts, six articles were removed because they were duplicates, leaving 101 articles to be read in full, according to the described selection steps. Finally, 32 articles were selected after excluding the ones that did not meet the pre-established eligibility criteria.

**Figure 1:**
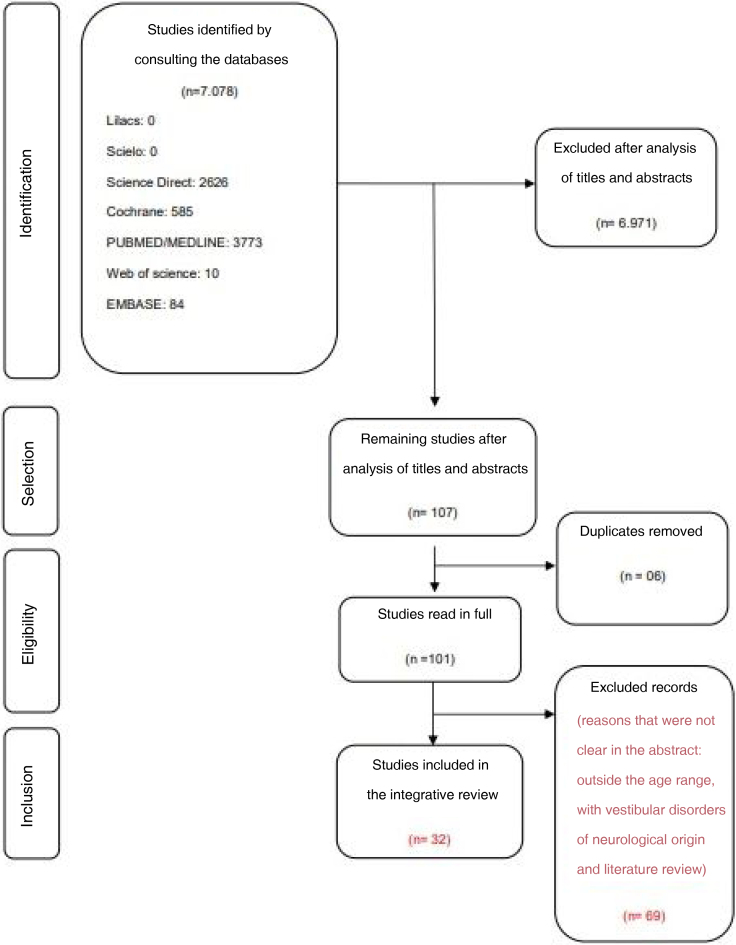
Flow diagram of article selection.

It is important to highlight that the exclusion of the 69 articles that occurred after full-text reading was due to lack of clarity or missing information in the abstract, being better described only throughout the text, such as: age group outside the one established by the study, articles involving children with vestibular disorders of neurological origin and literature reviews (Fig. 1).

After analyzing all the studies included in the integrative review, eight experimental studies were found, six of which were randomized clinical trials (level of evidence 2)^12^, ^13^, ^14^, ^15^, ^16^, ^17^ and two non-randomized ones (level of evidence 3)^18^, ^19^; eighteen observational studies (level of evidence 4)^5^, ^20^, ^21^, ^22^, ^23^, ^24^, ^25^, ^26^, ^27^, ^28^, ^29^, ^30^, ^31^, ^32^, ^33^, ^34^, ^35^; and six case studies (level of evidence 5),^36^, ^37^, ^38^, ^39^, ^40^, ^41^ according to the classification used.^11^

The included studies were carried out between the years 1969 and 2020, in the European,^5^, ^12^, ^13^, ^14^, ^18^, ^21^, ^22^^,^^27^, ^36^, ^41^ American,^15^, ^25^, ^26^, ^28^^,^^30^, ^32^, ^33^, ^34^, ^39^, ^40^ Asian^16^, ^17^, ^20^, ^23^^,^^24^, ^29^, ^31^, ^35^^,^^37^, ^38^ and African continents.^19^

The study participants’ age ranged from two days to twelve years old. The causes of referral of children for vestibular system assessment were diverse: hearing loss,^13^, ^16^, ^19^, ^20^^,^^23^, ^41^, ^42^ neonatal screening and/or maturation of the vestibular system,^5^, ^28^, ^29^, ^30^, ^32^, ^36^ learning deficit and/or poor school performance,^15^, ^32^, ^34^, ^38^^,^^39^ middle ear (ME) alterations and/or inner ear (IE) malformations,^17^, ^24^, ^28^, ^31^ at-risk and/or preterm infants,^12^, ^18^, ^22^ cytomegalovirus (CMV) infection,^25^, ^27^ attention deficit hyperactivity disorder (ADHD),^38^ assessment of the sacculo-collic reflex,^21^, ^37^ migraine,^14^ and benign paroxysmal vertigo in childhood.^35^

Vestibular assessment is the most appropriate way to identify early changes related to balance. Vestibular assessment was performed in all studies included in this review. The individuals who participated in this study were mostly children, aged between two and twelve years,^14^, ^15^, ^16^, ^19^, ^20^, ^23^, ^24^, ^25^, ^26^, ^27^, ^28^, ^31^, ^33^, ^34^, ^35^, ^38^^–^^41^ followed by infants aged between one and twenty-four months^5^, ^12^, ^13^, ^17^^,^^22^, ^27^, ^32^ and finally, newborns, with up to 29 days of life.^17^, ^21^, ^29^, ^30^^,^^36^, ^37^

Among the included studies, all performed some type of neurotological evaluation and some associated it with other tests. The vestibular function of the included studies was evaluated through the following tests: thirteen studies used rotary chair testing (RCT),^15^, ^18^, ^20^, ^23^^,^^25^, ^27^, ^28^, ^30^, ^31^, ^32^, ^38^, ^40^, ^41^ eight used caloric stimulation,^13^, ^15^, ^22^, ^23^, ^24^, ^25^, ^33^, ^36^ fourteen studies performed the cervical-vestibular evoked myogenic potential (cVEMP),^5^, ^12^, ^16^, ^17^^,^^19^, ^23^, ^25^, ^27^^,^^29^, ^35^, ^36^, ^37^, ^38^, ^40^ seven studies performed electronystagmography or vectoelectronystagmography (VECTO),^14^, ^20^, ^24^, ^28^^,^^32^, ^33^, ^36^ three analyzed the presence of spontaneous nystagmus,^19^, ^26^, ^39^ two studies used the video head impulse test (vHIT),^27^, ^41^ two studied the oculomotor function,^26^, ^30^ two studies performed the vestibulo-ocular reflex (VOR) assessment,^21^, ^38^ one study performed the spinal vestibule reflex (SVR) assessment ^14^ and one applied the following tests: Romberg, Unterberger, Fukuda and Dix-Halpike.^13^

Most studies associated vestibular assessment with other audiological tests: five performed an audiometry test on the participants,^14^, ^17^, ^33^, ^35^^,^^36^ six associated it with the auditory evoked potential (AEP),^5^, ^17^, ^20^, ^30^^,^^35^, ^36^ five applied the transient evoked otoacoustic emissions (TEOAE) and/or distortion product evoked otoacoustic emissions (DPOAE).^5^, ^17^, ^29^, ^36^^,^^37^ Finally, Moro reflex,^21^ ocular alignment,^21^ postural control,^40^ conditioned orientation reflex,^17^, ^20^ and gait and balance^19^, ^24^, ^26^ were also assessed. It is thought that this variation in the choice of exams was due to the participants’ different age groups, as well as the level of maturation of the peripheral and central auditory systems.

The aims of the studies were quite varied. The three most frequently used tests were the ones that used RCT, caloric stimulation and cVEMP.

Most studies aimed at using RCT as a type of vestibular assessment of the Vestibulo-Ocular Reflex (VOR) in children with Hearing Loss (HL),^15^, ^20^, ^23^, ^40^^,^^41^ maturation of the vestibular system,^28^, ^31^ infants and children with CMV infection,^25^, ^27^ vestibular function in newborns at neurological risk,^16^ IE malformations,^31^ and finally, children with ADHD.^38^

The use of caloric stimulation comes second, with some studies using bithermal stimulation – stimulation with hot and cold temperatures,^15^, ^25^, ^35^ some studies using only cold water^13^, ^22^ and others using ice-cold water.^23^, ^32^ Caloric stimulation has been used to assess the vestibular system in children with HL,^13^, ^15^, ^23^ at-risk infants,^22^ migraine,^15^ CMV infection,^25^ the assessment of the vestibular system maturation,^32^ and BPCV(benign paroxysmal vertigo of childhood ).^35^

All studies showed some ype of vestibular impairment through the results of caloric stimulation.^15^, ^22^, ^23^, ^25^^,^^32^ The vast majority showed some type of peripheral vestibular alteration, such as hyporeflexia and areflexia,^23^ uni- and bilateral hypoexcitability, and bilateral absence of excitability^22^ and weaker nystagmus reactions with cold stimulation.^13^

Finally, cVEMP, as a form of vestibular screening in newborns,^5^, ^36^ sacculo-collic reflex maturation,^37^ ADHD,^38^ and, finally, to verify vestibular function in at-risk infants (preterm, neurological disorders or CMV infection).^12^, ^25^, ^27^ Because it is an objective test and capable of assessing the vestibular system and sacculo-collic pathways, cVEMP has been considered a very promising test for the evaluation of the pediatric population, especially in relation to vestibular screening in newborns^17^ (Table 1).

**Table 1:** Description of studies included in the integrative literature review.

Author, year	Place	Objective	Study type	Age range	Sample	Reasons for referral	Hearing and/or vestibular complaints	Exams performed	Main conclusions
Adamović et al. (2010)^20^	Serbia	To assess the vestibular apparatus function and its pathways in newborns	Observational	Up to 29 days of life	100	Vestibular apparatus function and its pathways in newborns	NR	OAA, VOR and MR	It is suggested that vestibular pathways are normal after the child is born when the eyes are positioned in the midline with VOR and MR present.
Bernard et al. (2015)^24^	Montreal	To assess the incidence of vestibular disorders in children with CMV infection	Observational	Mean age: 34.7 months	52	CMV	NR	Bithermal caloric stimulation, vertical axis rotation, off-vertical axis rotation and cVEMP	Vestibular disorders are frequent and severe in children infected with CMV
Brookhouser et al. (1991)^29^	USA	To assess the vestibular development in the first year of life	Observational	Up to 06 months	65	Low- and high-risk full-term infants	NR	RCT, ABR and oculomotor test	The importance of establishing correlations between vestibular results and variables such as brainstem auditory results, birth history and postnatal evolution is highlighted.
Chen et al. (2007)^36^	Taiwan	To investigate the maturation of the sacculo-collic reflex in newborns	Case study	02 to 05 days of life de	40	Maturation of the sacculo-collic reflex in newborns	NR	DPOAE and cVEMP with head rotation	The VEMP in newborns can be easily recorded by the head rotation method. Prolonged or absent CVEMPs in newborns may reflect incomplete maturity of the sacculo-collic reflex pathway.
Dhondt et al. (2020)^26^	Belgium	To investigate the occurrence and characteristics of vestibular loss in children infected with CMV	Observational	6 months to 3 years	93	CMV	NR	vHIT, rotation test and cVEMP	Vestibular assessment should be part of the standard otorhinolaryngological follow-up in children with CMV, as CMV can impair vestibular function.
Ecevit et al. (2012)^11^	Turkey	Measure cVEMP in late preterm and full term infants and compare results	Randomized clinical trial	Between 4 and 8 week of life	34	Late preterm and full-term infants	NR	cVEMP	Abnormal cVEMP results may be related to a delay in the maturation of sacculo-collic pathways in late preterm infants
Erbek et al. (2007)^35^	Turkey	To determine cVEMP normality parameters for vestibular dysfunction in newborns	Case study	21 toa 28 days of life	24	Universal vestibular screening	NR	Tympanometry, EOA, ABR, cVEMP	cVEMP can be easily used for early assessment of vestibular dysfunction in newborns. It is suggested that services have their own normative standards, as values may differ according to technique and age.
Franco et al. (2007)^33^	Brazil	To study vestibular function in children with academic difficulties and associated symptoms.	Observational	7 to 12 years	50	Academic difficulties	Dizziness, nausea,	Audiological exams and VENG	All identified vestibular alterations were of peripheral origin and dizziness, nausea, reading and copying difficulties showed a statistically significant relationship between the studied variables.
Franco et al. (2008)^32^	Brazil	To study the vestibular function in children with poor academic performance	Observational	7 to 12 years	88	Poor academic performance	NR	Audiological exams and VENG	All vestibular alterations found had an irritative peripheral origin. There was a significant statistic association between vestibular alteration and school performance.
Golz et al. (1998)^23^	Israel	To determine the incidence of balance-related symptoms in children with ME effusion and to find out whether these symptoms resolved after insertion of ventilation tubes	Observational	4 to 9 years	136	ME effusion	NR	ENG and motor test	Balance-related symptoms found in young children may result from chronic ME effusion. These symptoms will resolve after evacuating the effusion and inserting ventilation tubes.
Horak et al. (1988)^14^	USA	To determine whether vestibular function can explain deficits in motor coordination in children with HL and learning deficits.	Randomized clinical trial	7 to 12 years	45	Hearing impairment and learning deficit	HL	RCT and caloric stimulation	Reduced or absent vestibular function in children with hearing impairment did not affect the development of motor proficiency, except in specific balance-related activities. Moreover, sensory organization deficits in the group with learning disability and in three of the hearing-impaired children were associated with generalized deficits in motor proficiency.
Kimura et al. (2018)^30^	Japan	To evaluate the relationship between vestibular function and gross motor development in children with IE malformations	Observational	3 months to 6 years	195	Children with IE malformation	HL	RCT	Structural malformations of the IE are associated with vestibular dysfunction and delayed gross motor development.
Kolkaila et al. (2015)^17^	Egypt	To assess vestibular function in children with OME	Non-randomized clinical trial	5 to 12 years	55	OME	CHL	Postural control and gait test, observation of spontaneous nystagmus and cVEMP.	Vestibular tests are valuable tools to detect vestibular dysfunction in children. Bone conduction cVEMPs are useful for the assessment of the vestibular system
Lotfi et al. (2017)^37^	Iran	To assess vestibular function in children with ADHD	Case study	7 to 12 years	60	ADHD	NR	cVEMP, VOR and RCT	Children with ADHD should undergo vestibular assessment, as high gains in VOR and decreased suppression capacity may result in symptoms of reading and writing difficulties, learning deficits, vertigo, and nausea.
Mailloux et al. (2014)^38^	USA	To analyze the post-rotatory nystagmus test in children with learning disabilities	Case study	02 months to 09 years	81	Learning difficulty	NR	PRN test	Given the importance of the vestibular system in early development, the PRN test should be considered for infants and young children.
Mäki-Torkko et al. (2005)^40^	Sweden	To report the vestibular evaluation of a consecutive series of children with profound SNHL.	Case study	12 to 90 months	6	SNHL	SNHL	Head impulse test and	The preliminary results show that the applied tests can be successfully performed in young children, as they provide useful information about vestibular function in pediatric patients.
Masuda et al. (2014)^18^	Japan	To assess the relationship between motor function acquisition and vestibular function in children with severe bilateral HL	Observational	03 months to 04 years	97	HL	HL	ASSR, AEP, COR, RCT and ENG	Vestibular function can be acquired with maturation of the vestibular system
Mierzwiński et al. (2000) ^13^	Poland	To describe vestibular function in children with migraine	Randomized clinical trial	Not reported	35	Migraine	NR	Threshold tonal audiometry, SVR, Romberg, Unterberger, Fukuda, Dix-Halpike and VENG tests	The pathological findings mainly suggest the central location of vestibular dysfunction in children with migraine, and the number of pathological VENG findings does not seem to be correlated with the type of migraine.
Mitchell et al. (1969)^31^	Canada	To determine the vestibular response in full-term neonates	Observational	Newborns up to 6 months	45	Normal full-term infants	NR	ENG, RCT and caloric stimulation	Absence of response is strongly suggestive of impaired vestibular function in all, except neonates in whom it is inconclusive.
Ornitz et al. (1979)^27^	USA	To describe the maturation process of the vestibular system	Observational	1 month to 11 years	460	Normal children in infancy and childhood	NR	RCT and ENG	The infant has a higher amplitude and beat velocity than an older child, with the velocity of the slow component being more significant in early childhood.
Pignataro et al. (1979)^16^	Italy	To check the vestibular response in infants at neurological risk	Non-randomized clinical trial	15 to 30 days of life	83	Neurological risks	NR	Rotatory test	The vestibular test appears as one of the most useful in the evaluation of the sensory sensitivity of infants at neurological risk.
Singh et al. (2012)^15^	India	To assess saccular function in severe to profound sensorineural hearing loss (SNHL)	Randomized clinical trial	4 to 12 years	15	SNHL	NR	_C_VEMP	Vestibular function plays an important role in gross motor development in children. Thus, speech therapists and otologists must recognize and understand vestibular dysfunction in children with hearing loss and be prepared to perform adequate assessments.
Rehagen et al. (2020)^25^	USA	To develop a screening protocol for children with OME	Observational	4 to 8 years	30	OME	CHL and SNHL	Tests of coordination and balance, oculomotor function and nystagmus	Children with CHL due to OME have more oculomotor abnormalities than their normal peers.
Sheykholesami et al. (2005)^19^	Japan	To demonstrate that vestibular function can be assessed by cVEMP in infants	Observational	01 to 12 months	12	EAC Atresia, Treacher-Collins Syndrome, and failure at the NHS	NR	COR, audiometry, OAE, ABR and _C_VEMP	cVEMP can be used as an objective test in infants and young children to explore the vestibular system and sacculo-collic pathways
Shinjo et al. (2007)^22^	Japan	To evaluate the vestibular function of babies and children with congenital and acquired deafness	Observational	31 to 97 months	20	Congenital and acquired deafness	HL	Cold water caloric test, RCT and _C_VEMP.	It is suggested that the presence of vestibular dysfunction is quite common in babies and young children with congenital and/or acquired deafness.
Valente et al. (2012)^39^		To report different vestibular cases in children	Case study	5 to 6 years	2	SNHL	SNHL	RCT, _C_VEMP and posturography	The early identification of vestibular alterations and intervention will help to optimize the child's function in daily life.
Vatovec et al. (2003)^21^	Slovenia	To assess the function of the vestibular apparatus	Observational	10 to 12 months	110	At-risk infants	NR	Frenzel goggles and caloric stimulation with cold water.	It is suggested that the vestibular system function should be tested in all children with HL, being very often found together with motor development delay.
Verrecchia et al. (2019)^5^	Sweden	To assess the feasibility of cVEMP together with the universal newborn hearing screening program	Observational	2.3 ± 1.9 months	50	Universal vestibular screening	NR	OAE, ABR, cVEMP	cVEMP has shown a high level of feasibility when used together with the regional newborn hearing screening program
Young et al. (2009)^28^	Taiwan	To investigate the maturation and development of the sacculo-collic reflex in newborns	Observational	2 to 13 days	45	Full-term infants	NR	DPOAE and _C_VEMP	cVEMP shows a prevalence of responses from the 5^th^ day onwards, maintaining a stable latency, and can be used to evaluate the sacculo-collic reflex
Zagólsk (2005)^12^	Poland	Evaluate high-risk hearing impaired children through caloric stimulation	Randomized clinical trial	3 to 6 months	58	High risk hearing impairment	HL	Caloric stimulation	Caloric stimulation is one of the few clinically-proven tests that assess the function of each vestibule separately in neonates. Its results represent the continuity of the VOR, starting in the lateral vestibular canal.
Zhang et al. (2012)^34^	China	Investigate the diagnostic value of the vestibular test and the high auditory stimulus rate of the ABR and the possible mechanism responsible for BPCV	Observational	3 to 12 years	56	Benign paroxysmal childhood vertigo	NR	Audiometry, ABR, _C_VEMP and bithermal caloric stimulation	Vascular mechanisms may be involved in the pathogenesis of benign paroxysmal childhood vertigo and there is strong evidence for its close relationship with migraine.

cVEMP, Cervical Vestibular Evoked Myogenic Potential; vHIT, Video Head Impulse Test; OAA, Ocular Alignment in waking hours; ABR, Auditory Brainstem Response; RCT, rotary chair testing; VOR, Vestibulo-Ocular Reflex Test; MR, Moro Reflex Test; OAE, Otoacoustic Emissions; DPOAE, Distortion Product Otoacoustic Emissions; ENG, Electronystagmography; VENG, Vectoelectronystagmography; PRN, Post-Rotary Nystagmus Test; ASSR, auditory steady-state response; AEP, Auditory Evoked Potential; COR, Conditioned Orientation Reflex; BPCV, Benign Paroxysmal Childhood Vertigo; ME, Middle Ear; IE, Inner Ear; OME, Otitis Media with Effusion; HL, Hearing Loss; SNHL, Sensorineural Hearing Loss; CHL, Conductive Hearing Loss; ADHD, Attention Deficit Hyperactivity Disorder; NB, Newborn; CMV, Cytomegalovirus; USA, United States of America; NR, Not Reported.

## Discussion

According to the studies included in the review, children considered at risk deserve special attention regarding the evaluation of the vestibular system. Infants at neurological risk may have altered vestibular sensitivity; thus, vestibular tests are considered very useful for this assessment.^18^, ^22^

Vestibular disorders are frequent and severe in children infected with CMV.^25^ These children may present with unilateral or bilateral, limited or extensive, stable or progressive, early or delayed vestibular alteration. As the vestibular function can deteriorate over time in children infected with CMV, the vestibular assessment should be part of the otorhinolaryngology follow-up in all children infected with CMV.^27^

Children with ADHD may have greater gains in VOR and poor ocular fixation reflex capacity when compared to typically developing children. Additionally, cerebellar dysfunction in these patients has also been documented in the literature, and the findings of the cVEMP and rotational chair test (RCT) for children with ADHD showed impaired vestibular function as the results, based on increased VOR gain values ​​and the decreased ocular fixation reflex capacity.^38^ Thus, the assessed study established a relationship between both ADHD and the vestibular system, and between poor school performance and possible vestibular alterations.^38^

Regarding preterm infants^12^, ^40^ and the vestibular system maturation,^37^ cVEMP may show prolonged and/or absent results. Prolonged and/or absent cVEMP findings reflect incomplete maturity of the sacculo-collic reflex. The myelination process is one of the main causes.^12^, ^37^, ^40^

Another relevant aspect is that some studies^20^, ^21^, ^24^ report that the development of the delayed motor aspect can be correlated with reduced vestibular function, in which the vestibular function can be acquired through to the maturation of the sensory hair cells of the vestibular system and the vestibular nerve of the inner ear (IE).^20^ The association between hearing loss and motor efficiency deficit has also been highlighted, which may require good balance in addition to abnormal responses in vestibular tests, such as cold caloric stimulation, RCT and cVEMP.^15^, ^23^

Although the literature mentions that the vestibular assessment can be difficult in infants and children, RCT is widely used, since it is one of the most frequently used tests, as it stimulates the semicircular canals and otoliths in both ears. Hence, it can detect responses even with a weak VOR.^21^

Finally, the association between structural IE malformations and reduced vestibular function and delayed motor development has also been highlighted,^20^ showing a higher incidence of reduced VOR in children with vestibular and semicircular canal malformations.^31^

Of note, the cerebellar connections with the vestibular system help to maintain VOR balance, contributing to postural balance and limb coordination.^42^ Moreover, VOR gain is interceded by the inferior olivary nucleus, in addition to being controlled by the cerebellum,^43^ which raises the hypothesis that central inhibition of vestibular function via the cerebellum may be deficient in children with ADHD, thus resulting in greater VOR gains.

Regarding the scenario of low school performance, dizziness, nausea, reading and copying difficulties showed a statistically significant relationship with the studied variables. Dizziness was the most common general complaint, reported by 36% of children and headache was the most common symptom, reported by 50% of participants in the school environment.^34^ A study reports that symptoms such as nausea, malaise and headache are common, demonstrating the involvement of alterations in the vestibular system.^44^

Regarding the vestibular alterations found in children with poor school performance, they had an irritative peripheral origin, showing a statistically significant relationship between vestibular alterations and school performance.^33^, ^34^ As balance is a vital neurological function in the process of maintaining adequate postures, being an essential factor in learning and a sign of neurological maturity. Vestibular alterations can compromise this school process, since learning is a complex and dynamic process, structured through the relationship between motor and perception skills, which, when cortically processed, give rise to cognition.^33^

A relevant aspect to consider is related to the vestibular findings of children with migraine. Findings from one study state that all children participating in the study who had migraine had abnormalities on VECTO vestibular tests, from calibration to caloric testing. The results and their analyses indicate that the functional status of the structures that make up the visual-ocular and vestibulo-ocular structures were altered in all migraine patients assessed in the study. Additionally, pathological findings mainly suggest a central location of vestibular dysfunction in children with migraine.^15^

Finally, regarding caloric stimulation; this is one of the few clinically proven tests that assess vestibular function separately in newborns. Its results represent the continuity of the VOR, starting in the lateral vestibular canal. It is worth noting that changes can be diagnosed in 20% to 70% of newborns who undergo the test and these changes are most often found in infants with perinatal pathology, multiple birth defects, and administration of aminoglycosides.^13^

## Conclusion

The most frequently used tests were RCT, caloric stimulation and cVEMP, because they are highly feasible tests that have the capacity to explore the vestibular system and maturation of the sacculo-collic pathway and reflexes, being the most common causes of referral for hearing loss assessment and vestibular screening.

## Conflicts of interest

The authors declare no conflicts of interest.
